# Hybrid total arch replacement via ministernotomy for Stanford type A aortic dissection

**DOI:** 10.3389/fcvm.2023.1231905

**Published:** 2023-10-18

**Authors:** Xing Liu, Xinyi Liu, Hong Yu, Yuehang Yang, Jiawei Shi, Qiang Zheng, Kan Wang, Fayuan Liu, Ping Li, Cheng Deng, Xingjian Hu, Long Wu, Huadong Li, Junwei Liu

**Affiliations:** ^1^ Department of Cardiovascular Surgery, Union Hospital, Tongji Medical College, Huazhong University of Science and Technology, Wuhan, China; ^2^ Department of Otorhinolaryngology, Union Hospital, Tongji Medical College, Huazhong University of Science and Technology, Wuhan, China

**Keywords:** Stanford type A aortic dissection, total arch repair, hybrid total arch repair, frozen elephant trunk, ministernotomy

## Abstract

**Background:**

Type A aortic dissection (TAAD) is a cardiovascular emergency condition with high mortality rate. Hybrid total aortic arch replacement using endovascular graft for the descending aorta repair results in favorable outcomes and has been recommended as an alternative procedure for the higher-risk category patients. Our institution started applying the upper ministernotomy incision technique for the hybrid procedures back in 2018.

**Methods:**

We collected patients who underwent hybrid total arch replacement (HTAR) via ministernotomy (96) and total arch replacement with frozen elephant trunk (TAR + FET) procedures (99), between 2018 and 2021. The baseline information, intraoperative and postoperative characteristics have been compared. Kaplan-Meier analysis was used for survival evaluation. Cox regression were applied to identify the independent predictor of mortality.

**Results:**

The baseline characteristics between the two patient groups were compared and found similar, except that RBC counts were higher (*p* = 0.038) and the ascending aorta diameter was smaller (*P* = 0.019) in the “HTAR” group relative to the “TAR + FET” group. The cardiopulmonary bypass time (*P* < 0.001), the aortic cross clamp time (*P* < 0.001), the operation duration (*P* = .029), ICU (*P* = 0.037) and postoperative hospital stay (*P* = 0.002) were shorter in the “HTAR” group. The “HTAR” group exhibited also significantly lower levels of intraoperative transfusion (all <0.001) characteristics than the “TAR + FET” group. The hospital mortality and 1-year mortality revealed similar patterns in both groups.

**Conclusion:**

HTAR via ministernotomy have similar short term prognosis, and also reduced the ICU and postoperative hospital stay. In all, The application of the ministernotomy technique in HTAR was safe and technically feasible and may benefit individual patients as well as hospitals in general.

## Introduction

Type A aortic dissection (TAAD) is a devastating disease with a high degree of mortality if not intervened promptly (1). TAAD, with its rapid progression, requires a multidisciplinary diagnosis in combination with a proper and timely surgical intervention. A poor prognosis for the disease is mostly associated with the aortic rupture and organ malperfusion (2, 3). Timely surgical reconstruction of aortic aneurysm and its dissection are the mainstays of the TAAD therapy. Total arch replacement with frozen elephant trunk (TAR + FET) has achieved desirable long-term outcomes and been widely used for TAAD treatment in China (4, 5). Nonetheless, the operative mortality of the disease remained high, owing to the inevitably large surgical invasions and long operation times (6).

While endovascular total arch repair is a new technique of limited utility (7), hybrid total arch replacement (HTAR) currently represents a more practical and extensive therapeutic strategy for TAAD (8). Initially, short and long-term outcomes of HTAR were reported by single-center clinical studies (9, 10), however, the small sample size in those studies has led to different experiences and conclusions. Subsequently, various institutions have successively utilized HART as one of the TAAD main treatments, and some even attempted to further improve the technique (8, 11).

Having benefited from the implementation and improvement of the TAAD repair procedures and the cerebral protection methods, surgeons have advocated for utilization of a minimally-invasive approach involving an upper ministernotomy, which had been used in cardiac surgery for nearly two decades (12), especially in aortic valve surgery, and, according to numerous studies, provided satisfactory outcomes (13, 14). Utilization of upper ministernotomy for the HTAR procedure has been initiated also at our institution. This study was aimed at determining if the use of upper ministernotomy for HTAR is safe for patients, as compared to the conventional TAR + FET intervention, and further advisable.

## Patients and method

### Study populations

**Figure 1:**
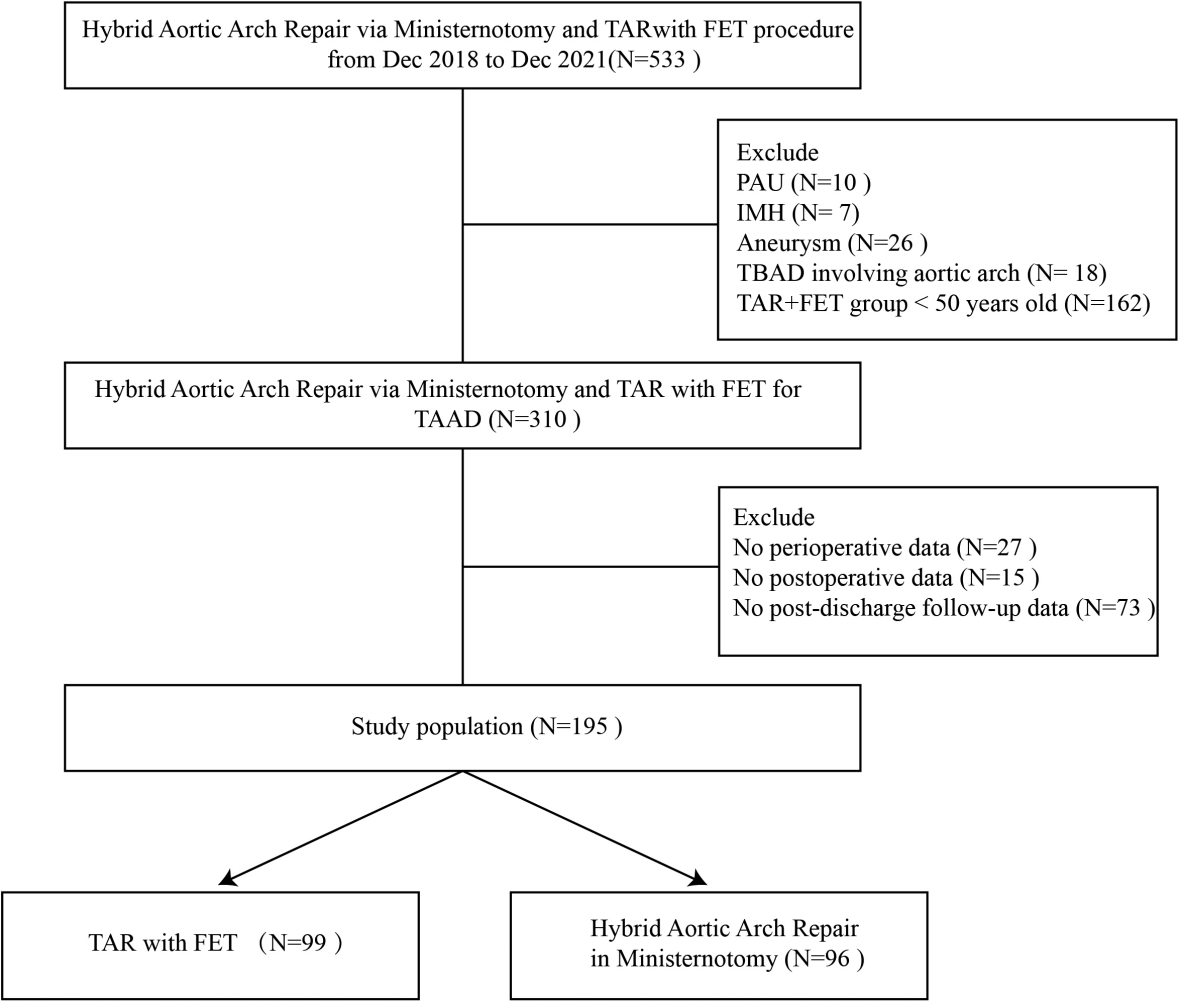
Flow diagram of the study cohort. Inclusion and exclusion criteria and population of this study. Five hundred thirty-three patients who underwent HTAR or TAR with FET procedure for TAAD from Dec 2018 to December 2021 were included. PAU, penetrating aortic ulcer; IMH, Intramural hematoma; TAAD, type A aortic dissection; TBAD, Stanford type B aortic dissection; FET, frozen elephant trunk.

We retrospectively included 195 patients who underwent surgery at Union Hospital between December 2018 and December 2021. All patients initially experienced sudden chest/back pain and were diagnosed with Stanford type A aortic dissection on the basis of computed tomography angiography (CTA). The morphology of the patients’ heart valves was assessed by using transthoracic echocardiography. Patients with intramural hematoma (IHM), aortic aneurysm, penetrating aortic ulcer (PAU), or Stanford type B aortic dissection (TBAD) were excluded from this study (Figure 1). All the patients included in this study underwent aortic dissection repair surgery. The patients were divided into two study groups based on the surgical method used in their treatment: 96 (49.2%) patients who underwent hybrid total arch repair via upper ministernotomy, and 99 (50.7%) who received a conventional TAR with FET. The “TAR + FET” group included only patients above age 50. At our center, Stanford Type A aortic dissection repair surgery is being performed by three staff chief physicians with similar surgical skills and experience. We collected medical history and examined clinical information, including test results, surgical records as well as the follow-up information for each patient enrolled in the study by using the Union Hosptial Records System. This study was approved by the ethics committee of Wuhan Union Hospital, Huazhong University of Science and Technology (UHCT22975) and complied with the World Medical Association Code of Ethics (Declaration of Helsinki) adopted in 1975.

### Surgical indication

In our hospital, hybrid total arch repair (HTAR) via ministernotomy and TAR + FET are currently the standard treatment options for Stanford type A aortic dissection. HTAR via ministernotomy is the most commonly used treatment of this disorder in the following circumstances: (i). in patients of old age (above 50) with multiple comorbid conditions who are at higher risk of hypothermic circulatory arrest (HCA); (ii) in patients with routine distal FET anastomosis that is a challenging condition when massive intimal tears or dissected pseudoluminas affect the distal portion of the descending aorta; (iii) in patients with high aesthetic wound requirements as HTAR via ministernotomy can shorten the period of wound healing.

### Surgical techniques

#### “TAR + FET” group

After induction of general anesthesia, the right axillary artery was cannulated for cardiopulmonary bypass (CPB) and ACP, and a standard median sternotomy was performed. Then CPB was carried out through the right axillary artery and right atrium, while the cooling process was initiated. When the patient was cooled down to 33°C or the heart suffered from a ventricular fibrillation, the ascending aorta was clamped. At this time, the surgical procedures in the aortic root were carried out. Then, the patient was continuously cooled down to about 25°C, at which circulatory arrest would be performed. The bilateral ACP was initiated through the left common carotid artery during the circulatory arrest. Meanwhile, the left subclavian, left common carotid and innominate arteries were clamped. The stented elephant trunk was inserted into the true lumen of the descending aorta, which was anastomosed to the distal end of the four-branched graft (Maquet M00202175728APO). As required, air was removed from the descending aorta after anastomosis. Blood perfusion of the lower body was initiated by the infusion limb of the four-branched graft. The left subclavian artery was anastomosed to one limb of the vascular graft. As a result, CPB gradually resumed to normal ﬂow, and the rewarming started. The left common carotid artery was anastomosed end-to-end with the innominate artery. Anastomosis of the proximal end was carried out during the rewarming step. When the lung was reventilated, the ascending aorta was reopened to resume the cardiac perfusion.

When the patients were cooled down to the temperatures below 28°C, a PH steady-state blood gas management was used, while an alpha steady-state blood gas management was applied when the temperature was above 28°C. The whole process of cooling and rewarming was carried out at a slow and uniform rate, in a step-by-step process. Following the operation, CPB was stopped when the blood gas analysis results were satisfactory.

#### “Hybrid total arch repair via ministernotomy” group

**Figure 2:**
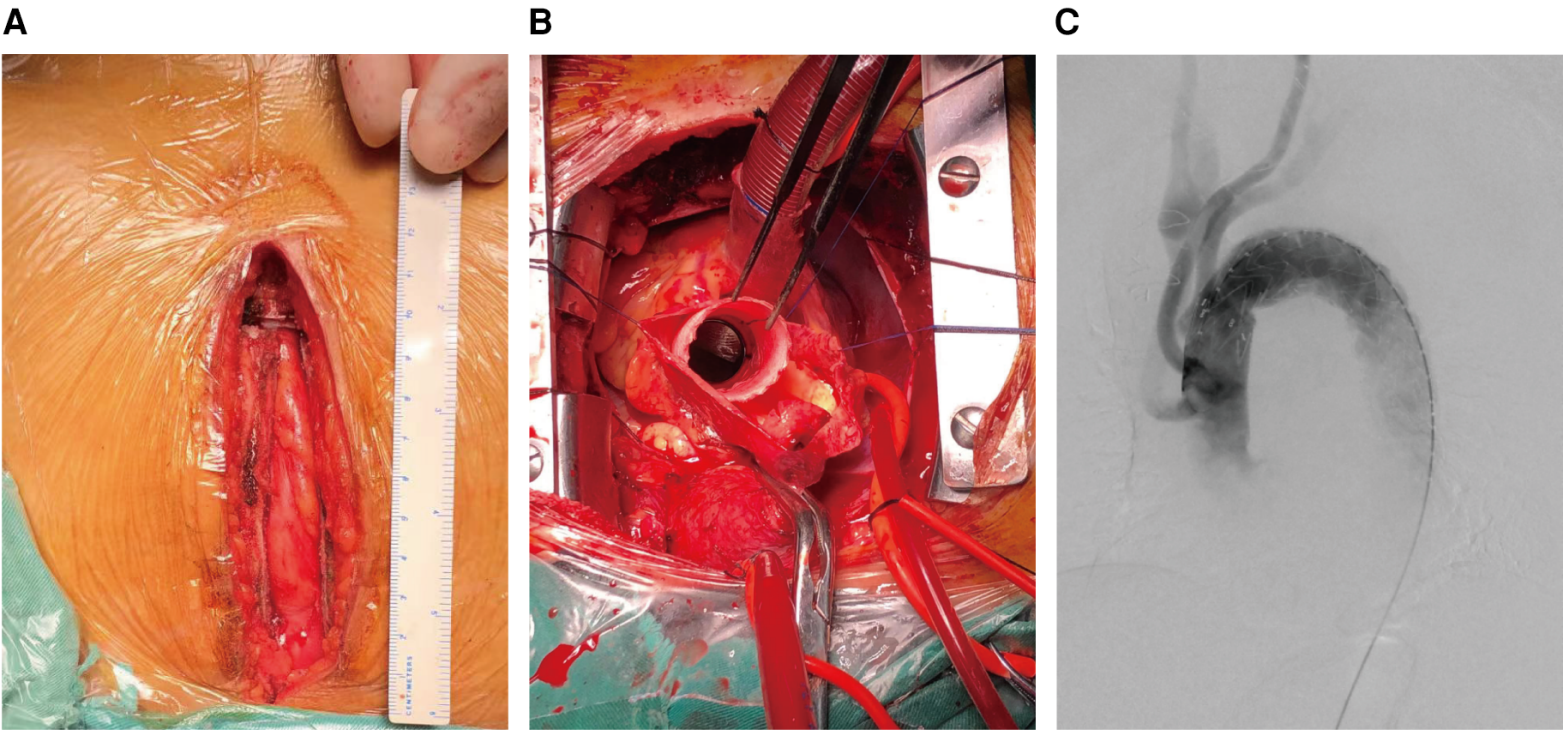
Incision of upper ministernotomy and HTAR technique. (**A**) Upper ministernotomy incision. (**B**) Intra-operative surgical filed of view: proximity of tetrafurcate vascular prosthesis graft was anastomosed to the sinotubular junction (aortic root has been repaired). (**C**) Endovascular portion: a stent graft implantation to exclude the entire lesioned aortic arch.

Our small-incision hybrid aortic repair is a single-stage procedure that was performed in a hybrid operating room and consisted of two phases: an open repair and an endovascular repair phase. The operation involved hybrid aortic arch repair without MHCA. In the open repair phase, a midline ministernotomy incision was made from the suprasternal fossa to the third intercostal (about 12–16 cm) (Figure 2A). Then the right femoral artery and the right atrium were used for cardiopulmonary bypass, and cooled to 32°C−28°C. The aortic cross-clamp placement was proximal to the opening of the anonymous artery and followed by the aortic root repair. Subsequently, a bilateral cerebral perfusion was initiated through the left common carotid artery and innominate artery catheterization. Meanwhile, the left subclavian carotid and innominate arteries were clamped. The aortic cross-clamp was used between the opening of the anonymous artery and the left common carotid artery. The aortic arch was transected proximal to the left common carotid artery. The distal end of the graft was then sutured end-to-end to the aortic arch, proximal to the left common carotid. An antegrade perfusion of the lower body was initiated by the branch of the artificial vessel, followed by rewarming and proximal anastomosis of the artificial vessel trunk (Figure 2B). The vessels in the parietal region were sequentially anastomosed with the left common artery, left subclavian artery, and anonymous artery. CPB was discontinued and wound hemostasis was achieved after protamine was administered to neutralize heparin. This is followed by endoluminal repair on digital subtraction angiography (DSA) (Figure 2C).

In the endovascular repair phase, the proximal stent was anchored to the artificial vessel to complete the arch repair. Angiography showed that there was no endoleak or contrast agent inside the false lumen of the thoracic aorta. A computed tomography performed prior to discharge showed aortic remodeling with complete thrombosis of the false lumen of the stented thoracic aorta. A postoperative transfer of the patients was made first to the intensive care unit (ICU) and then to a general ward, depending on the state of their recovery. After discharge, patients were advised to undergo a total aortic CTA examination in 3 months and 12 months after surgery, and annually thereafter.

### Outcome criteria

The primary outcome was a 1-year survival rate. The secondary outcome included such postoperative criteria as in-hospital mortality, postoperative complications, tracheotomy, extracorporeal membrane oxygenation (CRRT) secondary insertion pipe, tracheal intubation time, ICU stay, and postoperative hospital stay.

### Statistical analysis

Statistical analysis was performed by SPSS 25.0 software. Descriptive statistics was presented as frequency and percentage for categorical variables. For continuous variables, data was reported as mean ± SD or median with interquartile range after normal distribution testing. The non-normal distribution of the data necessitated the use of non-parametric tests. The Kaplan Meier survival estimate was calculated using Kaplan Meier analysis and Kaplan Meier curves were plotted. Cox univariable and multivariable regression analyses were performed and screened to show predictors of mortality. The variables used have been established in previous literature as predictors of the total arch replacement outcome.

## Results

### Baseline characteristics

The median age of patients in the “hybrid total arch replacement (HTAR) via ministernotomy” group was 60 (IQR:55–66) with 76 (79.2%) patients being males, whereas the median age of the patients in the “TAR + FET” group was 60 (IQR:53–67) with 78 (78.8%) being males. A moderate to severe aortic regurgitation occurred in 44 (45.8%) patients in HTAR and 42 (42.4%) in TAR + FET groups, respectively. RBC was statistically different between the two groups (4.18 vs. 3.97, *p* = 0.038). In addition, the results of the patients’ echocardiography analysis have shown that the ascending aorta diameter in the “HTAR via ministernotomy” group was smaller than that in the “TAR + FET” group (4.75 vs. 5.0, *P* = 0.019). Apart from the above, the baseline characteristics of both groups were identical, which is evident from Table 1. As shown in Table 2, there were no significant differences in coronary atherosclerosis disease (*P* = 0.268), hypertension (*P* = 0.154), or diabetes (*P* = 0.050) between the “HTAR via ministernotomy” and the “TAR + FET” groups. Moreover, there was no statistically significant difference between the two groups in terms of comorbidities.

**Table 1:** Baseline variables.

Variables	Hybrid total arch replacement (*n* = 96)	TAR + FET (*n* = 99)	*P* Value
Male sex, *n* (%)	76 (79.2)	78 (78.8)	0.948
Age, year, median (IQR)	60 (55–66)	60 (53–67)	0.549
BMI, kg/m^2^, mean ± SD	25.35 ± 8.76	25.69 ± 9.96	0.509
Smoking, *n* (%)	38.00 (39.60)	39.00 (39.40)	0.978
Alcohol drinking, *n* (%)	21.00 (21.90)	24.00 (24.20)	0.695
Systolic pressure, mmHg, mean ± SD	137.96 ± 21.91	135.20 ± 23.17	0.366
Diastolic pressure, mmHg, median (IQR)	80.00 (69.00–88.00)	80.00 (70.00–89.00)	0.988
Emergency operation, *n* (%)	59 (61.5)	69 (69.7)	0.226
Moderate to severe aortic regurgitation, *n* (%)	44.00 (45.80)	42.00 (42.40)	0.632
Hydropericardium, *n* (%)	31.00 (33.70)	26.00 (27.70)	0.372
RBC, 10^12^/L, median (IQR)	4.18 (3.65–4.50)	3.97 (3.36–4.45)	**0** **.** **038**
HCT, %, median (IQR)	37.10 (32.23–40.78)	36.30 (33.05–39.95)	0.419
Platelet, 10^9^/L, median (IQR)	152.50 (122.50–191.50)	146.50 (110.00–188.00)	0.234
WBC, g/l, median (IQR)	9.56 (7.72–12.13)	9.12 (6.55–12.21)	0.171
Ratio of lymphocytes, %, median (IQR)	9.15 (5.90–12.78)	10.10 (6.25–13.30)	0.444
Hemoglobin, g/L median (IQR)	124.50 (109.00–138.00)	121.00 (109.00–132.50)	0.286
Albumin, g/L, median (IQR)	35.75 (32.33–38.78)	37.00 (34.20–38.98)	0.153
Scr, μmol/L, median (IQR)	79.25 (65.40–109.30)	82.90 (68.90–125.95)	0.202
Bun, mmol/L, median (IQR)	6.76 (5.35–8.48)	7.16 (5.53–8.98)	0.400
Blood glucose, mmol/L, median (IQR)	6.30 (5.46–7.67)	6.21 (5.61–7.31)	0.883
Troponin I, ng/L, median (IQR)	27.80 (5.05–143.35)	16.60 (5.35–79.30)	0.257
INR, median (IQR)	1.10 (1.05–1.22)	1.10 (1.05–1.20)	0.590
APTT, s, median (IQR)	38.80 (34.40–41.33)	36.85 (34.65–42.18)	0.566
LVEF, %, median (IQR)	62.00 (60.00–65.00)	62.00 (60.00–65.00)	0.920
Aortic sinus diameter, cm, median (IQR)	3.90 (3.63–4.30)	4.05 (4.55–5.45)	0.355
Ascending aorta diameter, cm, median (IQR)	4.75 (4.30–5.13)	5.00 (4.55–5.45)	**0**.**019**
Aortic arch diameter, cm, median (IQR)	3.70 (3.40–4.13)	3.90 (3.50–4.30)	0.062
Distal diameter of the arch, cm, mean ± SD	3.47 ± 0.212	3.59 ± 0.173	0.071
Descending aorta diameter, cm, median (IQR)	3.90 (3.50–4.20)	3.90 (3.65–4.15)	0.635

BMI, body mass index; IQR, interquartile range; RBC, red blood cell; HCT, hematocrit; WBC, white blood cell; INR, international normalized ratio; APTT, activated partial thromboplastin time; LVEF, left ventricular ejection fraction.

Bold values indicated significance in statistical analysis.

**Table 2:** Comorbidities.

Variables	Hybrid total arch replacement (*n* = 96)	TAR + FET (*n* = 99)	*P* Value
History of heart surgery, *n* (%)	3 (3.1)	6 (7.1)	0.241
Hypertension, *n* (%)	72 (75.0)	65 (65.7)	0.154
Diabetes, *n* (%)	14 (14.6)	6 (6.1)	0.050
CAD, *n*, (%)	10 (10.4)	6 (6.1)	0.268
Pulmonary embolism, *n*, (%)	2 (2.1)	0 (0)	0.241
Stroke, *n*, (%)	4 (4.2)	8 (12.1)	0.111
CKD, *n* (%)	3 (3.1)	5 (5.1)	0.752
Chronic liver disease, *n*, (%)	3 (3.2)	0 (0)	0.223
Dialysis, *n* (%)	2 (2.1)	3 (3.0)	>0.999
Malperfusion, *n* (%)	3 (3.2)	5 (5.1)	0.753
Pericardial tamponade, *n* (%)	2 (2.1)	3 (3.0)	>0.999

CAD, coronary atherosclerosis disease; CKD, chronic kidney disease.

### Intraoperative data

Intraoperative data are detailed in Table 2. The CPB time (155 vs. 216 min, *P* < 0.001), the aortic cross clamp time (ACCT) (100 vs. 118.5 min, *P* < 0.001) and the operation duration (462.5 vs. 484.0 min, *P* = .029) times were shorter in the “HTAR via ministernotomy” group than in the “TAR + FET” group (Table 3). Besides, the “HTAR via ministernotomy” group exhibited also significantly lower values in intraoperative blood transfusion: RBC (6 vs. 9.5 units, *P* < 0.001), plasma (600 vs. 950 ml, *P* < 0.001), PLT (2 vs. 3 units, *P* < 0.001) compared with the “TAR + FET” group. Besides, the “HTAR via ministernotomy” and the “TAR + FET” groups revealed a difference with regard to the simultaneous surgical aortic root management by Bentall operation (13.5% vs. 37.4%, *P* < 0.001).

**Table 3:** Intraoperative variables.

Variables	Hybrid total arch replacement (*n* = 96)	TAR + FET (*n* = 99)	*P* Value
CPB time, min, median (IQR)	155.00 (129.00–190.00)	216.00 (171.50–263.75)	**<0** **.** **001**
ACCT, min, median (IQR)	100.00 (78.25–119.00)	118.50 (97.25–164.50)	**<0**.**001**
Operation duration, min, median (IQR)	462.50 (386.25–530.25)	484.00 (420.00–600.00)	**0**.**029**
SCPT, min, median (IQR)	0 (0.0)	16.00 (0–20.00)	**<0**.**001**
Intraoperative RBC transfusion, unit, median (IQR)	6.00 (4.13–7.88)	9.50 (7.25–11.50)	**<0**.**001**
Intraoperative plasma transfusion, ml, median (IQR)	600.00 (500.00–800.00)	950.00 (675.00–1,050.00)	**<0**.**001**
Intraoperative PLT transfusion, unit, median (IQR)	2 (2.00–3.00)	3 (3.00–4.00)	**<0**.**001**
Entry location envolvement of aortic root or sinotubular junction	8 (8.3)	21 (21.2)	**0**.**004**
Aortic root management, all, *n* (%)	17 (17.7)	40 (40.4)	**<0**.**001**
David, *n* (%)	0 (0.0)	2 (2.0)	0.491
Bentall, *n* (%)	13 (13.5)	37 (37.4)	**<0**.**001**
Wheat, *n* (%)	4 (4.2)	1 (1.0)	0.347
CABG, *n* (%)	9 (9.4)	6 (6.1)	0.385

CPB, cardiopulmonary bypass, ACCT, aortic cross clamp time; SCPT, selective cerebral perfusion time; CABG, coronary artery bypass grafting.

Bold values indicated significance in statistical analysis.

### Short-term postoperative outcomes

There were no significant differences in the outcomes of post-surgical continued treatment such as ECMO, secondary thoracotomy operation, tracheotomy, tracheal intubation time, secondary insertion pipe, and CRRT (*P* > 0.05). However, the length of the patients’ ICU stay (129 vs. 153 h, *P* = 0.037) and the length of postoperative hospital stay (20 vs. 24 days, *P* = 0.002) were shorter for the “HTAR via ministernotomy” group relative to the “TAR + FET” group. As far as the postoperative complications are concerned, no difference between the groups was noted, as from the 12 patients who died during the immediate postoperative period, 6 were from the “HTAR via ministernotomy” group and 6 from the “TAR + FET” group (6.3% vs. 6.1%, P = 0.941), while no patients exhibited a postoperative stent endoleak or stent displacement. The percentage of postoperative pulmonary complications, however, was lower in the “HTAR via ministernotomy” group than in the “TAR + FET” group (60.4% vs. 83.7%, *P* < 0.001). Nonetheless, there were no significant differences in postoperative pericardial effusion (*P* = 0.360), postoperative neurological complications (*P* = 0.421), and postoperative renal or liver dysfunctions (*P* = 0.539).

### Survival analysis

**Figure 3:**
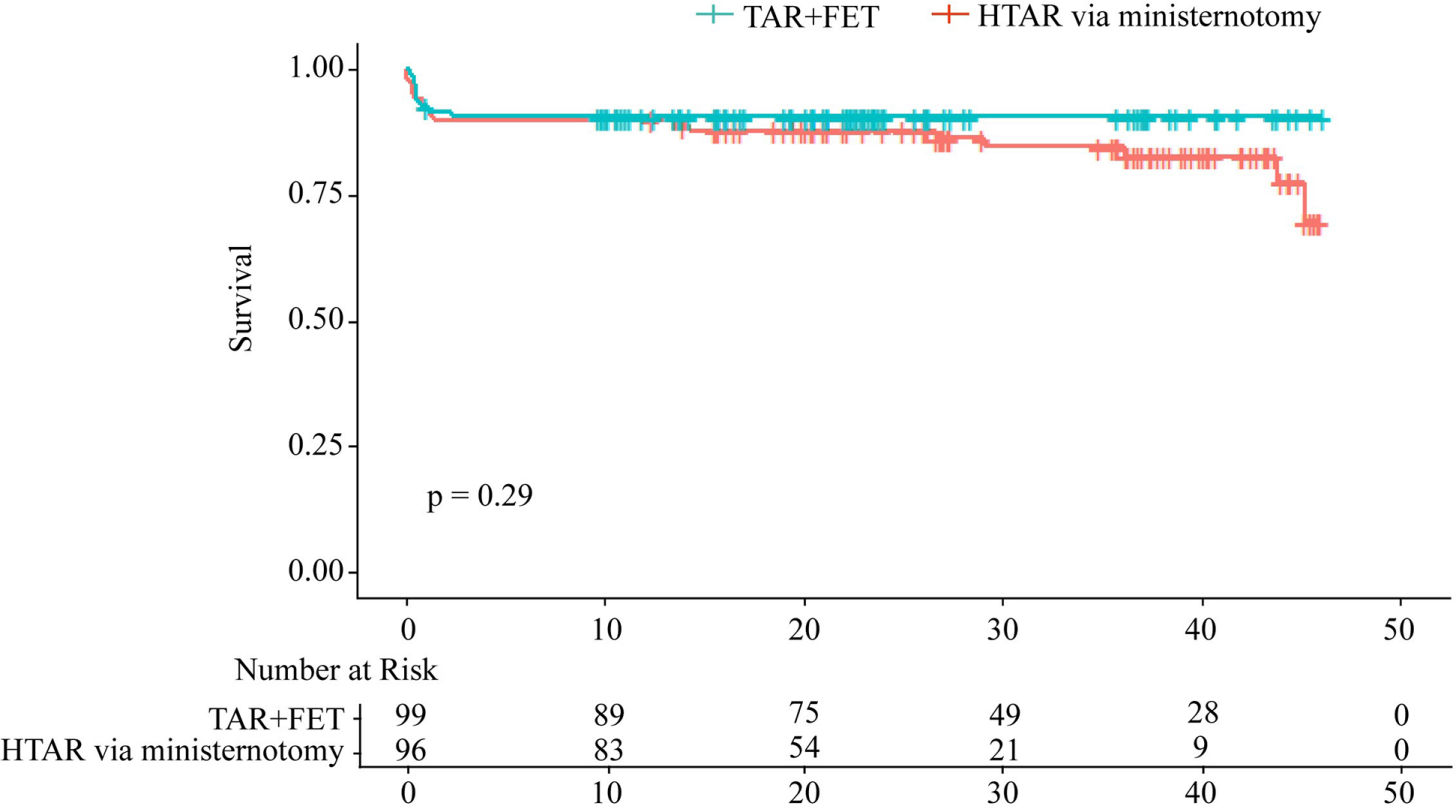
Kaplan–Meier analysis for overall survival stratified by HTAR via ministernotomy and TAR + FET, and log-rank test showed no significant difference between two group (*P* = 0.29).

**Figure 4:**
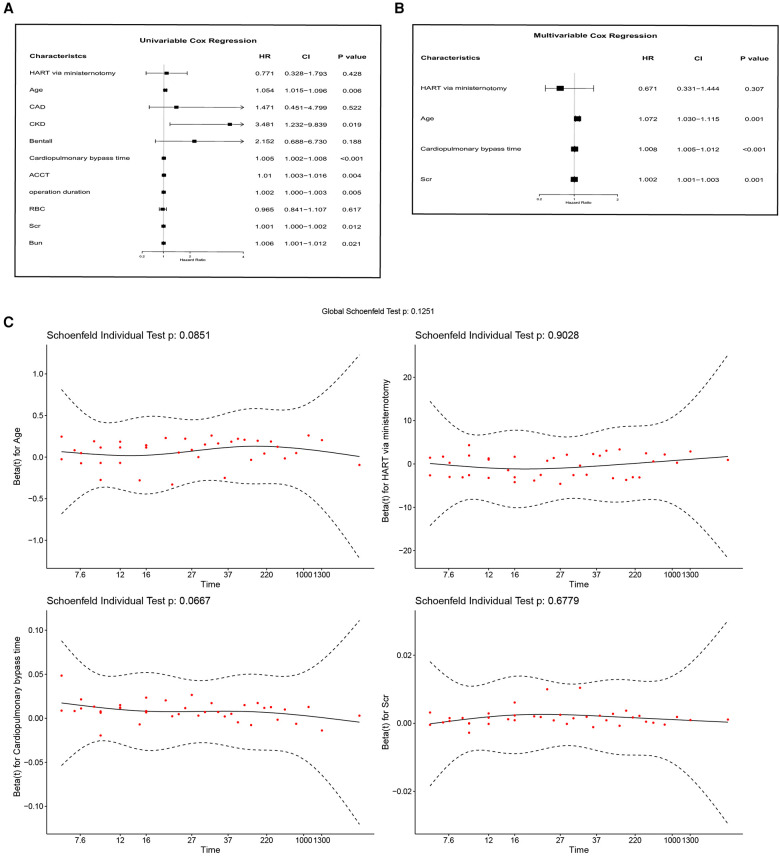
*Cox* proportional hazards regression models. (**A**) Univariable *Cox* regression analysis; (**B**) multivariable *Cox* regression analysis. (**C**) Schoenfeld residuals analysis. HR, hazard ratio; CI, confidence interval.

All patients were followed up until October 10th, 2022, and the median follow up time was 29.0 (16.5–40.7) months for the “TAR + FET” group and 20.8 (10.3–27.3) months for the “HTAR via ministernotomy” group, respectively. In Kaplan–Meier survival analysis, no significant differences were found between the “HTAR via ministernotomy” group and the “TAR + FET” group (*p* = 0.29) (Figure 3). Separate analyses of hospital mortality (5.2% vs. 5.1%, *p* = 0.960) and 1-year mortality (10.4% vs. 11.1%, *p* = 0.876) revealed similar patterns (Table 4). The variables were selected according to the previous studies and the baseline variables that have a significant bias between the two groups for univariable Cox analysis (Figure 4A). On the basis of Cox multivariable regression analysis, we found that the application of ministernotomy for HTAR was safe [HR 0.671 (0.331–1.444); *p* = 0.307] (Figure 4B). Furthermore, older age [HR 1.072 (1.030–1.115); *p* = 0.001], cardiopulmonary bypass time [HR 1.008(1.005–1.012); *p* < 0.001] and Scr [HR 1.002 (1.001–1.003); *p* = 0.001] represented significant independent predictors of mortality in both univariable and multivariable models. The Schoenfeld residuals analysis was performed and showed no significance (Figure 4C).

**Table 4:** Short-term postoperative outcomes.

Variables	Hybrid total arch replacement (*n* = 96)	TAR + FET (*n* = 99)	*P* Value
Secondary thoracotomy operation, *n* (%)	2 (2.1)	1 (1.0)	0.979
Tracheotomy, *n* (%)	4 (4.2)	10 (10.1)	0.109
Secondary insertion pipe, *n* (%)	5 (5.2)	13 (13.1)	0.056
Tracheal intubation time, hour, median (IQR)	67.00 (41.25–118.25)	80.00 (54.50–155.50)	0.085
ICU stay, hour, median (IQR)	129.00 (88.50–209.00)	153.00 (111.00–260.25)	0.037
Postoperative hospital stay, day, median (IQR)	20.00 (16.00–24.75)	24.00 (18.00–35.00)	**0** **.** **002**
CRRT, *n* (%)	14 (14.6)	11 (11.1)	0.468
Postoperative pericardial effusion, *n* (%)	60 (63.2)	68 (69.4)	0.360
Postoperative paraplegia, *n* (%)	3 (3.1)	2 (2.0)	0.972
Postoperative pulmonary complications, *n* (%)	58 (60.4)	82 (83.7)	**<0**.**001**
Postoperative neurological complications, *n* (%)	5 (5.2)	8 (8.1)	0.421
Postoperative renal dysfunction, *n* (%)	30 (28.6)	28 (29.4)	0.862
Postoperative liver dysfunction, *n* (%)	40 (49.4)	41 (50.6)	0.971
False lumen patency persisted, *n* (%)	5 (5.2)	8 (8.1)	0.421
Hospital mortality, *n* (%)	5 (5.2)	5 (5.1)	0.960
1-year mortality, *n* (%)	10 (10.4)	11 (11.1)	0.876

PLT, platelets; IABP, intra-aortic balloon pump; ECMO, extracorporeal membrane oxygenation; CRRT, continuous renal replacement therapy.

Bold values indicated significance in statistical analysis.

## Conclusion

HTAR via ministernotomy have similar short term prognosis, and also reduced the ICU and postoperative hospital stay. In all, The application of the ministernotomy technique in HTAR was safe and technically feasible and may benefit individual patients as well as hospitals in general.

## Discussion

This retrospective study on acute TAAD patients has led to the following findings: primarily, the application of the ministernotomy for HTAR was safe and technically feasible compared with the traditional aortic repair. Second, the differences in intraoperative variables have indicated that the HTAR via ministernotomy procedure has shortened duration of the operation, CPB, and ACC, as well as reduced the intraoperatively needed blood transfusion volume of RBC, plasma, and PLT. More importantly, the ministernotomy for HTAR procedure *per se* was not an independent risk factor for the patient mortality. However, older age, the cardiopulmonary bypass time, and the Scr level remained independent risk factors after adjusting for covariates.

As the technology improving the sensitivity of diagnostic for TAAD advances, more and more patients receive timely surgical interventions before the aortic rupture occurs. A prompt surgical treatment along with improved surgical techniques and perioperative patient care make the TAAD disease increasingly curable. Despite the advances in all the above fields, the operative and perioperative morbidity and mortality for TAAD remains high. In this regard, the goal of clinical TAAD treatment has not only been to improve the patients’ survival, but also to obtain a better long-term prognosis for those patients. Since the traditional total aortic repair surgery was still a highly-invasive and risky procedure, the endovascular treatment of the thoracic aortic disease is emerging as a less invasive alternative to open surgery (15, 16). With the steadily increasing use of EAVR, it has been the mainstay of the descending aortic disease treatment (17, 18). Since the HTAR approach was first implemented in 2000 by J.A. Macierewicz et al. (19), many cardiovascular centers applied it for over a decade and their data show that HTAR has achieved desirable early and long-term clinical outcomes (9, 20). Our method of HTAR via upper ministernotomy procedure has similar indications as the conventional HTAR approach. Our data also indicated a favorable outcome after adjusting for age, which is the factor of the greatest bias from the TAR group (10). A meta-analysis of 38 studies reported that the hospital mortality associated with HTAR for TAAD was 5.5% lower than that in the case of traditional total arch repair (11). While a 12-year retrospective study involving the HTAR treatment of 209 patients in China demonstrated an early mortality rate of 10.0% (9), our study's early mortality associated with HTAR via ministernotomy was only 5.2%, (5.1% in the “TAR + FET” group), and a 1-year mortality was 10.4% (11.1% for the “TAR + FET” group). The rate of early mortality associated with our ministernotomy for HTAR technique was similar to that resulting from the conventional invasion of HTAR procedure. The two procedures compared in our study significantly differed in such characteristics, as post-operative in-hospital time (20 vs. 24; *p* = 0.002) and post-operative pulmonary complications (60.4% vs. 83.7%; *p* < 0.001). Except for the comparable short-term outcomes, the two groups exhibited significant differences in intraoperative variables, in line with the previous studies demonstrating that the HTAR procedure decreased the time of CPB, ACC, as well as the operation duration time (20). Besides, the use of HTAR via ministernotomy reduced the intraoperatively needed blood transfusion volume.

It is noteworthy that “HTAR” reported by some earlier studies was referred to as a “TAR with FET” procedure. Actually, TAR with FET that is also called Sun's procedure, has been widely used for the TAAD treatment in China for over 20 years (21). In our institution HTAR has been utilized as an alternative option for TAR with FET, especially for the high-risk patient category. In this case, there was a notable discrepancy in patients’ age between the two groups. We excluded patients under the age of 50 from the “TAR with FET” treatment group also because the indication age for the HTAR treatment in our institution was 50 years of age and over. Patients’ age has been previously reported as an independent predictor for mortality and used as such also in our study [HR 1.072 (1.030–1.115); *p* = 0.001]. This, to some extent, decreased the risk of potential bias. Besides, our results were consistent with those of an earlier study, reporting that, after propensity-score matching (PSM), the early mortality and post-operational complication rates in the “HTAR” group were not significantly different from the “TAR + FET” group (10).

The upper ministernotomy approach was first implemented for aortic valve operations in 1996, and since 1997 its use was extended to more complex cardiac surgery procedures (13, 22, 23). A surgical department from Italy with 11 years of experience in applying upper ministernotomy for the ascending aorta procedures, has found that this technique can reduce the postoperative bleeding and thereby the number of transfused RBC units in patients, as well as reduce their hospital stay (14). These outcomes were similar to those of our study and demonstrated that the utilization of upper ministernotomy provides substantial clinical advantages (24, 25). Both the satisfactory clinical outcomes and patients’ requests facilitated application of ministernotomy for HTAR. To date the usage of this technique has been limited to a few large cardiovascular centers. More time is obviously needed for this technique to be widely and more commonly used in complex cardiac surgeries. A center in China has applied upper ministernotomy for the conventional TAR with FET procedure, but their results showed no difference in ICU and total hospital stay (26). Besides, they selected low-risk patients for this minimally-invasive surgery, and this made the above study different from ours. Our purpose thus was to further decrease the risk of infection and improve the poor surgical wound healing, since the HTAR procedure was indicated for the high-risk category patients. To our delight, our study revealed that the use of HTAR via ministernotomy has led to less post-operative pulmonary complications, which would mainly involve a pulmonary infection and excessive or moderate pleural effusions. In conclusion, practicing upper ministernotomy for HTAR in our institution proved safe and feasible, alleviated both patients’ and hospital's burden and provided a minimal invasiveness to the surgical process.

## Study limitations

There are few limitations to this study. First, this was a single-center based retrospective observational study, which can only provide a limited clinical and statistical information. A multi-center study with larger sample size may be needed. Second, we didn't include a perfect control group because, since 2018, almost all the patients with HTAR indication received surgery using the upper ministernotomy approach at our institution. However, the data of HTAR via ministernotomy in this study was discussed and compared with previous studies of conventional invasion of HTAR procedure obove. Third, since the use of this approach in our institution has not been long enough, the data on the long-term outcomes of this study are not yet available, and the patients from our study should still be followed up in the future.

## Data Availability

The original contributions presented in the study are included in the article/Supplementary Material, further inquiries can be directed to the corresponding authors.
